# Effects of Restoration Time on Microbial Diversity in Rhizosphere and Non-Rhizosphere Soil of *Bothriochloa ischaemum*

**DOI:** 10.3390/ijerph15102155

**Published:** 2018-09-30

**Authors:** Tong Jia, Miaowen Cao, Ruihong Wang

**Affiliations:** 1 Institute of Loess Plateau, Shanxi University, Taiyuan 030006, China; caomw1993@163.com (M.C.); rui_hong_w@163.com (R.W.); 2 Technology Development Department, Shanxi Xinghuacun Fenjiu Group Wine Industry Development Zone Limited by Share Ltd., Fenyang 032205, China

**Keywords:** *Bothriochloa ischaemum*, rhizosphere, bacterial diversity, fungal composition, restoration time, soil enzyme activity

## Abstract

There is well-documented evidence that shows phytoremediation and restoration methods affect physical and chemical properties, enzyme activities, and microbial communities of soil. In this study, we investigated the response of soil microbial communities to restoration time. We found that arsenic content decreased gradually as restoration progressed. Total carbon (C) in shoots and total nitrogen (N) in roots of *B. ischaemum* both exhibited increasing trends with an increase in restoration time. The transfer factor of chromium was negatively correlated to C in shoots and positively correlated to sulfur in roots. Additionally, the transfer factor of lead had a remarkably positive correlation to the C/N ratio of roots. For soil enzymes, total N in soil was positively correlated to catalase and urease but negatively correlated to sucrose. Moreover, bulk soil bacterial composition was positively correlated to catalase, sucrase and phosphatase while fungal diversity was positively correlated to sucrose. This study found that restoration time plays the most significant role in bacterial and fungal composition and bacterial diversity, but it has no effect on fungal diversity in rhizosphere and non-rhizosphere soil. In addition, the driving factors of microbial composition and diversity varied in rhizosphere and non-rhizosphere soil among the different restoration time treatments.

## 1. Introduction

Heavy metal soil pollution is one of the most critical issues related to mining processes. Heavy metal pollution in mining areas primarily derives from dust, coal gangue, and acid waste water produced during mining processes. Additionally, these heavy metals enter the soil through several means and pathway, such as wind erosion-derived dust, migration, deposition, and precipitate leaching. The drainage of mine tailings can cause arsenic (As) pollution as well as other highly toxic substances in the surrounding soil, while also decreasing plant mineral elements and soil pH conditions unsuitable for plant growth [1]. Microbes, soil enzymes and unique microbial groups are the three most important soil components during restoration processes in mining areas, which all play a vital role in nutrient cycling while improving soil fertility and decontamination. Based on plant characteristics that can either tolerate or accumulate certain pollutants, phytoremediation can remedy, absorb, and beneficiate pollutants by means of plants and their co-existing microbial systems. Plant-microbial remediation technology has gained considerable attention in recent years [2,3,4,5,6]. Plants and their associative microbial rhizosphere communities can symbiotically consolidate their survival strategies toward restoration, which can influence the form and bioavailability of contaminants as well as promote the absorption of pollutants by plants.

The soil environment supports soil microbial subsistence. Even though various soil nutrient types provide sources of energy for microbial use, the transformation and production of nutrient substrates depend on microbes and soil enzymes. Soil enzymes are important components of the ecosystem production and the core of the soil ecosystem structure and function. They participate in all soil biochemical reactions, promote the transformation of carbon (C), nitrogen (N), phosphorus (P), and other elements for cycling, and encourage energy metabolism and pollutant decontamination [7,8,9]. In addition, soil enzymes work in conjunction with soil microbes to promote metabolic processes in soil and soil enzyme activity can be used as an activity index of soil quality and an evaluation index of soil fertility [10]. Therefore, the study of soil enzymes can help us to better understand interactions among plants, soil, and microorganisms, as well as reveal the flow of energy and nutrients in the plant-soil-microbial continuum.

Soil enzymes such as urease, sucrose, and catalase are more sensitive, which can reflect the toxic effect of heavy metals on soil microbial activity [11]. As a crucial source of soil enzymes, soil microbes can produce extracellular enzymes and play a critical role in regulating the soil ecosystem by maintaining the stability of the material cycle and purifying soil. Additionally, soil microbes are sensitive to heavy metal pollution and reflect soil quality, which is an important biological index to evaluate soil quality [12]. To date, most research has focused on soil microbial and enzyme activities under heavy metal pollution. Li Wei et al. [13] have reported on the effects *Chlorophytum comosum* on the quantity of soil microbial and enzyme activity under Zn contaminated, which found the abundance of soil bacteria, fungi and actinomycetes as well as the activities of catalase, sucrase, urease, and phosphatase in the experimental group were significantly higher than those in the control group. It indicated that *C. comosum* has an obvious remediation effect on soil heavy metal of Zn contaminated. Gao Yang et al. [14] studied the effect of maize growth on the microbial community structure and the enzyme activity under single and complex pollution of Cd and Pb, which displayed that planting maize could improve soil respiration intensity under heavy metal pollution and reduce the effect of Cd and Pb on phosphatase and urease.

The microbial communities of the rhizosphere are important driving forces and the main participant in biogeochemical cycles of various life-providing elements in terrestrial ecosystems and they also promote the absorption and utilization of plants by converting organic nutrients into inorganic nutrients. Moreover, the rhizosphere provides a place and a medium for microbial metabolism to take place. Therefore, interactions among the rhizosphere, plants, and microorganisms maintain the ecological function of the soil ecosystem [15]. In addition, rhizosphere microorganisms play significant roles in plant growth and development, nutrient acquisition, yields and disease and insect defense mechanisms while photosynthetic products from plants provide both a substrate and energy for rhizosphere microbial subsistence [16]. At the same time, microbial diversity in the rhizosphere is abundant. Many studies have reported that the surface of the plant root system with its high microbial diversity is one of the most complex microbial community collectives on the planet. Moreover, cell density of various microbes that colonize the root surface is much higher than that of plants and the number of genes from rhizosphere microorganisms far exceeds that of plant genes [17]. Plants influence the structure of the rhizosphere microbe community by secreting root exudates. Additionally, there is also a certain degree of dependence and specificity between plants and rhizosphere microorganisms. Conversely, however, structural changes in rhizosphere microbe communities affect plant root exudates, material circulation, energy flow, and information transmission within the soil system, which affects the growth, development and diversity of plant communities [18,19].

Much of the relevant research to date has focused on the characteristics related to the succession of vegetation communities after restoration in mining areas and the effect of different vegetation restoration methods on soil properties, enzyme activities, and microbial communities. It has been reported that soil C and N content is significantly affected by different vegetation restoration methods and durations [20]. Soil enzyme activities and microbial properties have increasingly been used as indicators of soil quality in the evaluation of reclamation efforts [21,22]. It has been reported that soil enzymes and microbes have been used to assess the success rate of reclamation methods in surface coal mines treated with various restoration methods in the Loess Plateau [23]. However, relatively few studies have been conducted on soil microbes that are more sensitive to ecological restoration and enzyme activities. That being said, a few studies have been conducted on rhizosphere and non-rhizosphere microbial communities in mining areas under heavy metal contamination.

This study was conducted in the eighteenth river tailings of the Northern Copper Mine in Yuanqu County, Shanxi Province, China. In each sub-dam, different vegetation types have established themselves following the commencement of phytoremediation and the subsequent restoration phases. Among these vegetation types, *Bothriochloa ischaemum* is an edificator within most plant communities [24]. *B. ischaemum* is a perennial grass species in the grass family (Poaceae), genus *Bothriochloa kuntze*, and belongs to the thermophilic and mesoxerophyte plant group. In this study, we investigated the response of soil bacterial and fungal communities in rhizosphere and non-rhizosphere soil to restoration time in a copper tailings dam using the denaturing gradient gel electrophoresis (DGGE) technique. We addressed the following questions: (1) What are the characteristics and influencing factors of soil enzyme activities for different restoration times within a heavy metal polluted environment? (2) Do rhizosphere and bulk soil communities differ in different sub-dams? (3) What are the driving factors for rhizosphere and non-rhizosphere soil microbial composition and diversity in sub-dams for different restoration times?

## 2. Materials and Methods

### 2.1. Site Description and Soil Sampling

The eighteenth river tailings of the Northern Copper Mine (35°15′~35°17′ N, 118°38′~111°39′ E) was constructed in 1969 in the southern region of Shanxi Province, China. Currently, the eighteenth river tailings are composed of 14 sub-dams with a stack height of 84 m and a texture ratio of 1:6 [25]. It is under the influence of a continental monsoon climate with four distinct seasons where the annual mean temperature is 14 °C, annual precipitation is approximately 780 mm, and frost-free days are greater than 200 day/year [26].

In July 2015, we selected five sub-dams (S536, S531, S529, S525, and S523) under different restoration stages (19, 23, 27, 31, and 35 years, respectively) for sampling [25]. For each sub-dam, we randomly collected shoot and root samples from *B. ischaemum* in five 1 m × 1 m sample plots following an S-shaped curve. At the same time, we collected rhizosphere and non-rhizosphere soil samples from each plot. Visible roots and residue were removed prior to homogenizing the soil fraction of each sample. Fresh soil samples were sifted through a 2 mm sieve and divided into two subsamples. One subsample was stored at 48 °C to determine physiochemical properties while the other was stored at 20 °C prior to DNA extraction.

### 2.2. Soil Chemical Properties and Enzyme Activities

Soil pH was measured after shaking in a soil-water (1:2.5 m/v) suspension for 30 min. Soil water content (SWC) was measured gravimetrically. Soil particle size (PS) was measured using the Mastersizer 3000 laser diffraction particle size analyzer (Malvern Panalytical Ltd., Malvern, UK). Total plant and soil C, N, and sulfur (S) content was measured by using an elemental analyzer (vario EL/MACRO cube, Elementar, Hanau, Germany). Nitrate nitrogen (NO_3_^−^-N), ammonium nitrogen (NH_4_^+^-N) and nitrite nitrogen (NO_2_^−^-N) were determined by using an automated discrete analyzer (CleverChem 380, DeChem-Tech. GmbH, Hamburg, Germany). Heavy metal (As, Cd, copper (Cu), Pb and Zn) sample concentrations were measured using the ICP-AES (iCAP 6000, Thermo Fisher, Cambridge, UK). Additionally, soil sucrase was measured by using 3,5-Dinitrosalicylic acid colorimetry. Urease was measured using phenol-sodium hypochlorite calorimetry. Catalase was measured by using potassium permanganate titration, and phosphatase was measured by using the disodium phenyl phosphate calorimetric method [27].

### 2.3. DNA Extraction, Polymerase Chain Reaction and Denaturing Gradient Gel Electrophoresis

Total soil DNA was extracted using the E.Z.N.A. Soil DNA Kit (Omega Bio-tek, Inc., Norcross, GA, USA). The structure of the bacterial and fungal communities was evaluated using the DGGE technique as follows: Fragments of *16S rRNA* genes (the V3 region) were amplified by a polymerase chain reaction (PCR) using primers 341F (5′-CGC CCG CCG CGC GCG GCG GGC GGG GCG GGG GCA CGG GGG GCC TAC GGG AGG CAG CAG-3′) and 534R (5′-ATT ACC GCG GCT GCT GG-3′). Fragments of *18S rRNA* genes (the V4 region) were amplified by PCR using the primers FUNG-GC (5′-CGC CCG CCG CGC CCC GCG CCC GGC CCG CCG CCC CCG CCC CAT TCC CCG TTA CCC GTT G-3′) and NS1 (5′-GTA GTCA TAT GCT TGT CTC-3′). The DGGE runs were performed using a DCode system (GelDoc XR, Biorad, Hercules, CA, USA).

### 2.4. Statistical Analysis

Significant differences between sub-dam variables were analyzed by one-way analysis of variance (ANOVA) and Duncan’s new multiple range test. The Pearson correlation coefficient was used to analyze the relationship between soil physicochemical properties and enzyme activities. Furthermore, DGGE image analysis of the band profiles were carried out by using Quantity One 1-D Analysis software version 4.6.2 (Bio-Rad Laboratories, Inc., Hercules, CA, USA), which detects bands and quantifies the relative DNA concentration. The number of distinct DGGE bands was imported into SPSS version 20.0 (IBM, Chicago, IL, USA) to calculate the Shannon–Wiener index (*H’*), the Margalef’s richness index (*dMa*), an evenness index (*En*), species richness (*S*), and the Simpson’s Diversity Index (*D*) [28]. The Mantel test was used to test relationships between microbial composition and diversity, soil and root variables, and restoration years and transfer factors. Statistical analyses were performed using the Canoco 5.0and Sigma Plot 12.5.

## 3. Results

### 3.1. Plant and Soil Characteristics of B. ischaemum for the Different Restoration Times

With the exception of NH_4_^+^-N, we observed significant differences in soil physical and chemical properties among the different restoration times (Table 1). NH_4_^+^-N showed a decreasing trend with an increase in recovery time and its content was the highest in the S529 sub-dam. As restoration time increased, we found a significant difference in As content (Table 2). Moreover, As content gradually decreased as recovery time increased and, between the recovery times investigated, the S536 sub-dam had the highest As content overall (25.44 mg/L).

**Figure 1:**
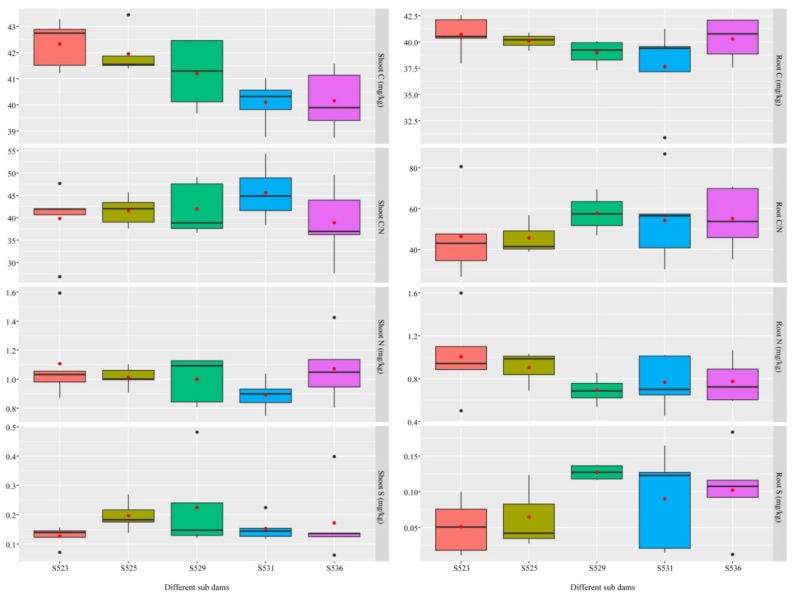
Total nitrogen (N), total carbon (C), ratio of carbon and nitrogen (C/N), and total sulphur (S) in shoot and root of *B. ischaemum*. Different-colored boxes represent different sub-dams (red, S523, olivine, S525, green, S529, blue, S531, pink, S536).

This study observed increasing trends in total stem C content and total root N content of *B. ischaemum* with an increase in restoration time (Figure 1). Results from the Mantel test showed that the physiological characteristics of aboveground plant components (r = 0.1396, *p* = 0.046) and roots (r = 0.1394, *p* = 0.032) of *B. ischaemum* were significantly correlated to the restoration time.

There were no significant changes in heavy metal transfer factors among the different restoration times (Table 2). Correlation analysis showed that TF-Cr was significantly negatively correlated to the total C content in stems and positively correlated to the total S content in roots. Additionally, TF-Pb was significantly positively correlated to the C/N ratio (Table 3).

### 3.2. Correlation betweenPlant Physicochemical Properties and Soil Characteristics

The Mantel test showed that enzyme activities were significantly correlated to C content in roots (Mantel statistic: r = 0.5796, *p* = 0.007) and Zn content in soil (r = 0.4012, *p* = 0.017). Additionally, soil total nitrogen was highly significantly and positively correlated to catalase and urease, but highly significant negatively correlated to sucrase (Table 3). However, we found no significant correlations between phosphatase and soil physicochemical properties, but we observed a significant negative correlation between phosphatase and total C and S in *B. ischaemum* (Table 3).

Furthermore, arsenic was significantly negatively correlated to total N and C in soil, positive correlations between soil C/N and As and Cu were also observed. Similarly, we found a significant positive correlation between SWC and Cu (Table 3), which indicated that high SWC content is not beneficial to Cu absorption. Moreover, there was a positively correlation between Zn and soil NO_3_^−^-N and a negative correlation between Zn in soil and C in roots (Table 3). One possible reason for this is that Zn is a necessary element for plant growth and the storage of C in roots is conductive to Zn absorption from the soil. Additionally, we found a significant negative correlation between N in stems and Pb in soil (Table 3), which indicated that an increase in plant nutrition could lead to a reduction in Pb content in soil.

### 3.3. Effects of Restoration Time and the Rhizosphere on Microbial Diversity and Gene Abundance

Restoration time had a significant impact on the diversity and gene abundance of bacteria and fungi while the rhizosphere mainly affected the diversity of fungi as well as the gene abundance of bacteria and fungi. The interaction between restoration time and the rhizosphere had a marked impact on the bacterial diversity but not on fungal diversity (Table 4).

**Figure 2:**
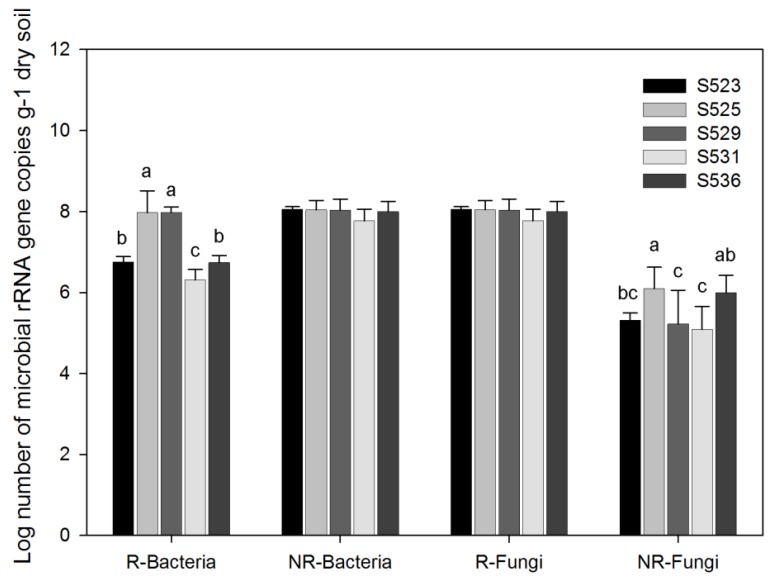
Abundance of rhizosphere (R) and non-rhizosphere (NR) soil bacteria and fungi *rRNA* genes in different sub-dams. Points show the means of five replicates, and vertical bars show standard errors. The different letters (a, b and c) indicate that the means are significantly different among restored sub-dams (*p* < 0.05) with the Duncan test.

This study found highly and significantly different gene copy numbers between bacteria in rhizosphere and fungi in non-rhizosphere soil (Figure 2). In addition, the gene copy numbers of rhizosphere bacteria in the S525 and S529 sub-dams were significantly higher than those in the other three sub-dams. As shown in Figure 2, the numbers of fungal copies in non-rhizosphere soil of the S525 and S536 sub-dams were significantly higher than those in the other sub-dams.

**Figure 3:**
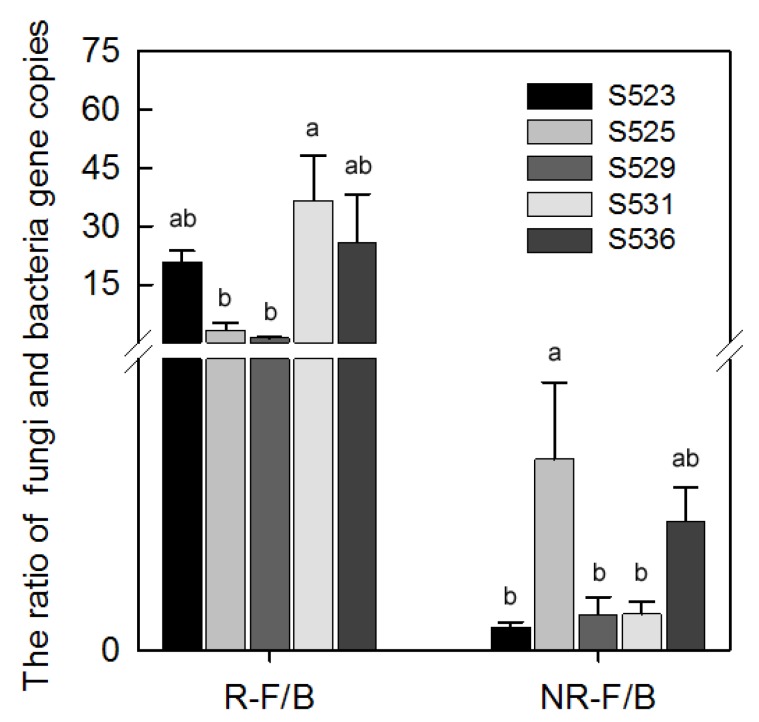
The ratio of bacteria and fungi gene copies (B/F) of rhizosphere (R) and non-rhizosphere (NR) soil in different sub-dams. The different letters (a, b and c) indicate that the means are significantly different among restored sub-dams (*p* < 0.05) with the Duncan test.

The ratio of fungi to bacteria (F/B) is shown in Figure 3. Among the different restoration years (times), we found a significant difference in the F/B ratio in the rhizosphere. Moreover, the F/B ratio in the rhizosphere of the S531 sub-dam was significantly higher than those of the S525 and S529 sub-dams. Additionally, the F/B ratio of non-rhizosphere soil microbes in the S525 sub-dam was significantly higher than those of the S523, S529, and S531 sub-dams (Figure 3).

### 3.4. Relationship between Soil Enzyme Activity and Microbial Diversity

Enzyme activity was significantly correlated to bacterial composition in the rhizosphere (r = 0.2016, *p* = 0.029) and non-rhizosphere (r = 0.3603, *p* = 0.001) according to results from the Mantel test. Among these, the composition and diversity of bacteria and fungi in the rhizosphere showed no significant correlation to soil enzyme activity. Conversely, the composition of bacteria in non-rhizosphere soil was significantly positively correlated to catalase, sucrase, and phosphatase. Fungal diversity in non-rhizosphere soil was also positively correlated to sucrose (Table 5). The reason for this could be that microorganisms in non-rhizosphere soil are more involved in the process of enzyme synthesis compared to the rhizosphere and rhizosphere microbes are mainly affected by secondary metabolites, such as root secretions.

### 3.5. Driving Factors of Microbial Composition and Diversity in Rhizosphere and Non-Rhizosphere Soil

According to results from the Mantel test, restoration time played the most significant role in the composition of bacteria and fungi and in bacterial diversity, but restoration time had no effect on fungal diversity in rhizosphere and non-rhizosphere soil (Table 6).

**Figure 4:**
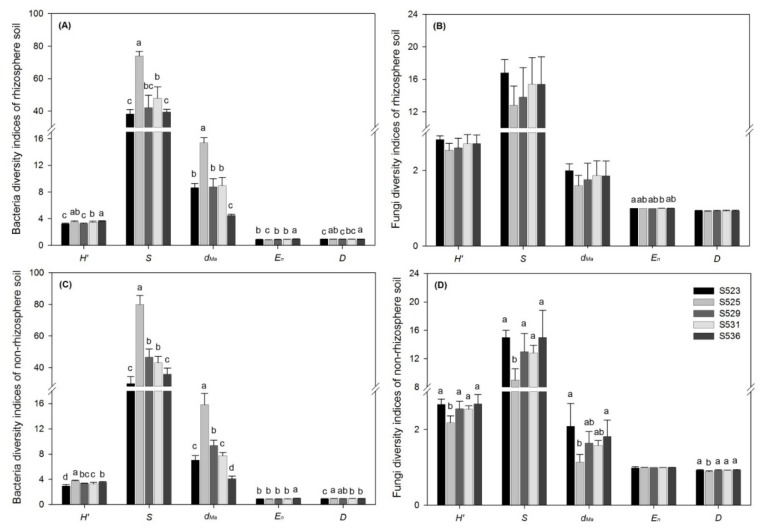
Shannon-Wiener index (*H’*), Species richness (*S*), Margalef (*d_Ma_*), Evenness (*E_n_*), and Simpson index (*D*) of rhizosphere (**A**,**B**) and non-rhizosphere (**C**,**D**) soil bacteria (**A**,**C**) and fungi (**B**,**D**) community in different sub-dams. Different letters (a, b and c) indicate significant differences, according to Duncan’s test (*p* < 0.05).

The results from bacterial evenness from the Margalef’s richness index (*dMa*) and bacterial richness from the species richness test (*S*) in the S525 sub-dam were significantly higher than in the other sub-dams in rhizosphere and non-rhizosphere soil and results from the evenness index (*En*) in the S536 sub-dam were significantly higher than in the other sub-dams (Figure 4A,C). Findings on rhizosphere bacterial diversity from the Shannon–Wiener index (*H’*) and the Simpson’s Diversity Index (*D*) in the S536 and S525 sub-dams were significantly higher than in the S523 and S529 sub-dams (Figure 4A). In addition, findings from the Shannon–Wiener index and the Simpson’s Diversity Index on non-rhizosphere soil bacteria in the S525 sub-dam were higher than in the S523, S531, and S536 sub-dams (Figure 4C).

The results from the evenness index in the S523 sub-dam were significantly higher than in the S531 sub-dam for fungi (Figure 4B). Conversely, diversity significantly differed among the different restoration times with the exception of results from the evenness index on fungi in non-rhizosphere soil. At the same time, results from the Shannon–Wiener index, species richness, and the Simpson’s Diversity Index on non-rhizosphere fungi in the S525 sub-dam were significantly lower than in the other sub-dams (Figure 4D), which was opposite to the results observed for non-rhizosphere bacteria. This may be due to the different survival strategies of bacteria and fungi in soil.

According to results from the Mantel test, rhizosphere bacteria composition was significantly correlated to the Zn transfer factor (r = 0.2294, *p* = 0.008) and Pb content in roots (r = 0.1653, *p =* 0.019). Bacterial diversity was significantly correlated to soil C (r = 0.2345, *p* = 0.008), but the composition and diversity of non-rhizosphere bacteria did not correlate to the other factors.

Fungal composition was significantly correlated to soil C (rhizosphere: r = 0.2037, *p* = 0.015, non-rhizosphere: r = 0.2037, *p* = 0.02) and pH (rhizosphere: r = 0.2152, *p* = 0.002, non-rhizosphere: r = 0.2152, *p* = 0.002). Rhizosphere fungal diversity was significantly correlated to NO_3_^−^-N (r = 0.1613, *p* = 0.035) and non-rhizosphere fungal diversity was significantly correlated to PS (r = 0.3897, *p* = 0.011).

### 3.6. Correlation between Soil Properties and Microbial Diversity of Rhizosphere and Non-Rhizosphere Soil for the Different Restoration Times

**Figure 5:**
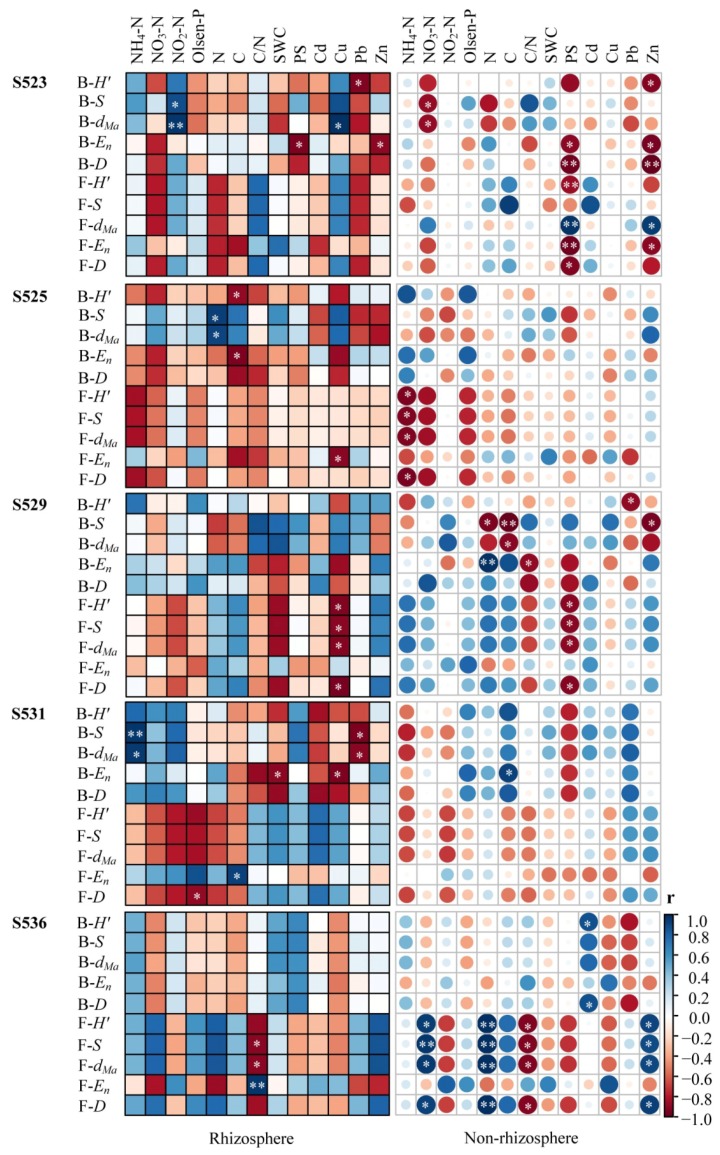
The Pearson correlations between soil properties and Shannon-Wiener index (*H’*), species richness (*S*), Margalef (*d_Ma_*), evenness (*E_n_*) and the Simpson index (*D*) of rhizosphere and non-rhizosphere soil bacteria (B) and fungi (F) community in different sub-dams. Soil properties include total nitrogen (N), total carbon (C), total sulphur (S), and a ratio of carbon and nitrogen (C/N). ** Correlation is significant at the 0.01 level (2-tailed). * Correlation is significant at the 0.05 level (2-tailed).

The driving factors of microbial composition and diversity varied in rhizosphere and non-rhizosphere soil among the different restoration times (Figure 5). Results from the species richness test and the Margalef’s richness index on bacteria in the rhizosphere were significantly and positively correlated to NO_3_^−^-N and the evenness index was negatively correlated to PS and Zn. Fungal diversity in non-rhizosphere soil was significantly correlated to PS in the S523 sub-dam (Figure 5). Results from the species richness test and the Margalef’s richness index on bacteria in the rhizosphere were significantly and negatively correlated to N in the S525 sub-dam. The results from the Shannon–Wiener index and the evenness index on bacteria in the rhizosphere were negatively correlated to C and fungal diversity in non-rhizosphere soil was negatively correlated to NH_4_^+^-N (Figure 5).

Furthermore, fungal diversity in the rhizosphere was negatively correlated to soil Cu and negatively correlated to PS in non-rhizosphere soil of the S529 sub-dam (Figure 5). Results from the species richness test and the Margalef’s richness index on bacteria in the rhizosphere were significantly positively correlated to NH_4_^+^-N and negatively correlated to Pb in the S531 sub-dam (Figure 5). Additionally, fungal diversity in the rhizosphere was significantly correlated to the C/N ratio and the soil fungi in the non-rhizosphere was significantly correlated to NO_3_^−^-N, total N, the C/N ratio and Zn (Figure 5).

## 4. Discussion

### 4.1. Soil Enzyme Activity and Driving Factors

Soil enzymes are one of the most active soil organic ecosystem components and they are involved in all biochemical processes within the soil environment. Additionally, soil enzymes are not only the agent for which soil organic transformations take place. They are also an active pool of plant nutrients [29]. Soil enzyme activity is also widely used as a biological index to evaluate soil health.

The results from this study showed that heavy metal pollution in soil could affect the function of soil biology, the diversity of the microbial community [30], and the transformation of C, N, P, and S in soil [31]. This study found significant differences in soil nutrients and enzyme activities [32]. This finding is in agreement with a previous study that showed that there were significant differences in soil physicochemical properties over different reclamation years [25]. Catalase activity indicates the degree of soil humus and organic matter accumulation and its relevance to organic matter content. Catalase is often used as a soil fertility indicator [33].

Urease can catalyze and hydrolyze urea into ammonia or ammonia ions. Additionally, among enzymes, urease has the most significant effect on the soil N cycle [34]. The results from our study consistently showed that soil N was highly and significantly positively correlated to catalase and urease (Table 3). Mantel test results showed that there was a significant correlation between enzyme activity and Zn in soil (r = 0.4012, *p* = 0.017). A possible explanation for this is that the soil substrate, or soil substrates, where soil protein chelation has taken place, become more complex in the presence of heavy metals or they react with enzyme substrates and produce complex reactions that inhibit soil enzyme activities [35]. Among the soil enzyme types, dehydrogenase, urease, and phosphatase are particularly important for nutrient transformation to take place in plants [36,37]. Furthermore, plant roots also directly affect soil enzyme activities [38]. Our study found that As content decreased gradually, total C and N in the stems of *B. ischaemum* increased with an increase in restoration time. There was a significant negative correlation between phosphatase activity and total C and S in the roots of *B. ischaemum* (Table 3). The effect of plant nutrients on enzyme activities could potentially be explained by two factors: (1) through substrate induction likely caused by the aboveground vegetation litter input, which appears to influence soil enzyme activities [39] and (2) through varied plant root stimulation on enzyme activities resulting from their different effects on microbial activity and exudate richness production in substrates [40].

Furthermore, this study also verified that bacterial composition was positively correlated to catalase, sucrase, and phosphatase and fungal diversity was also positively correlated to sucrase in non-rhizosphere soil. Nevertheless, there was no correlation between bacterial and fungal composition and diversity and soil enzyme activity in the rhizosphere (Table 5). To explain such findings, it could be reasoned that soil enzymes acted catalytically with bioactive substances secreted by microorganisms, animals, and plants and they were then released through animal and plant residue that decomposed in the soil [41]. Soil enzymes mainly derived from microorganisms in non-rhizosphere soil, which include intracellular enzymes existing in living cells and extracellular enzymes that exist in soil solutions or are adsorbed on the surface of soil particles [42,43]. Hence, microorganisms in non-rhizosphere soil have significantly greater effects on soil enzyme activity when compared to rhizosphere soil.

### 4.2. Microbial Gene Abundance and Diversity in Rhizosphere and Non-Rhizosphere Soil

Environmental factors and soil conditions such as soil moisture, directly influence microbial community structure and activity [44]. Furthermore, our study indicated that restoration time had a significant effect on diversity and gene abundance of both bacteria and fungi while specific micro-ecosystems in the rhizosphere mainly affected fungal diversity (Table 4). In addition, the number of bacterial gene copies in rhizosphere soil and fungi in non-rhizosphere soil was significantly different among the sub-dams investigated in this study (Figure 2). This was consistent with findings by Li et al. showed that reclamation scenarios determined soil microbial abundance, diversity, and composition [32]. Moreover, microbes in the rhizosphere of the different sub-dams could differ significantly. Therefore, the restoration time of different sub-dams and the specific micro-ecosystem in the rhizosphere could also have an effect on microbial community composition and diversity.

Micro-ecosystems in the rhizosphere, being the most active part of the global biochemical cycle, are the bond that bind plants, soil, and microbes and it is the core region of biogeochemical cycling that organically connects the atmosphere, biosphere, and pedosphere [45,46]. Our study revealed that the number of rhizosphere bacterial gene copies in the S525 and S529 sub-dams was higher than the other three sub-dams investigated and the number of non-rhizosphere fungal gene copies in the S525 and S529 sub-dams was higher than the other sub-dams investigated (Figure 2). A probable reason for this was that bacteria play a crucial role in ecological stability during the initial restoration phase [47] and a previous study reported that forest succession promoted a dominated soil fungal community [48]. In a pampa biome, the fungal community structure was more affected by land-use type [49]. These findings suggested that soil bacteria and fungi play different ecological roles [32].

Bacterial diversity results from the species richness and Margalef’s richness indices in rhizosphere and non-rhizosphere soil of the S525 sub-dam were significantly higher than the other sub-dams while bacterial diversity results from the Shannon–Wiener and Simpson indices in non-rhizosphere soil of the S525 sub-dam were higher than the S523, S531, and S536 sub-dams (Figure 4C). However, the opposite was true for fungi in non-rhizosphere soil. Fungal diversity results from the Shannon–Wiener, species richness, and Simpson indices in non-rhizosphere soil of the S525 sub-dam were significantly lower than in the other sub-dams (Figure 4D). A potential reason for this is that bacteria and fungi possess different survival strategies in soil. Furthermore, the ratios of fungal to bacterial (F/B) biomass also correlated to restoration processes due to the fact that elevated ratios could indicate the amount and composition of litter that enters the soil given that fungi are the dominant decomposers of plant cell wall polymers in the litter [50]. There were significant differences in F/B ratios in the rhizosphere under different restoration times and the F/B ratio in the S531 sub-dam was significantly higher than in the rhizosphere of the S525 and S529 sub-dams. Moreover, F/B ratios of microbes in non-rhizosphere soil showed that the S525 sub-dam was significantly higher than the S523, S529, and S531 sub-dams (Figure 3). We found that the S531 and S525 sub-dams had the highest F/B ratios in rhizosphere and non-rhizosphere soil, respectively (Figure 3), which could be indicative of a significant increase in fungal abundance under greater plant diversity [51].

### 4.3. Driving Factors of Soil Microbial Community Composition and Structure in Rhizosphere and Non-Rhizosphere Soil under Different Restoration Times

The composition of the soil microbial community is influenced by many environmental factors. Soil properties strongly influence microbial composition and microbial functionality within the soil system [52] such as soil moisture [53], agrotype [54], soil physicochemical properties [55], vegetation type and diversity [56], heavy metal type and content [57], and restoration time [58]. The species evenness of bacteria in the rhizosphere of the S523 sub-dam was negatively correlated to PS; fungal diversity in non-rhizosphere soil was significantly correlated to PS. Fungal diversity in non-rhizosphere soil of the S529 sub-dam was mainly negatively correlated to PS (Figure 5). These results implied that these microbial communities were closely correlated to soil physicochemical properties. Previous studies have also supported the hypothesis that states PS has a significant effect on microbial community structure [24].

In this study, sub-dam soil was alkaline (pH = 7.88–8.11). Mantel test results demonstrated that rhizosphere bacteria were significantly correlated to the Zn transfer factor (r = 0.2294, *p* = 0.008) and Pb content in roots (r = 0.1653, *p* = 0.019). Additionally, rhizosphere fungal diversity in the S529 sub-dam had a significant negative correlation to Cu in soil (Figure 5). This indicated that the soil microbial community of the different sub-dams was influenced by different driving factors even though both soil types (i.e., rhizosphere and non-rhizosphere soil) shared a similar pH profile (pH = alkaline) [59]. On the other hand, the nature of the contaminant (e.g., heavy metal pollution) could affect microbial diversity and abundance [60].

Soil pH is one of the most important and basic soil properties and acts as an index of soil formation and fertilizer cultivation, which have a significant influence on the form and availability of soil nutrients, soil physical properties, chemical properties, microbial activity and plant growth and development [61]. We found that there was a significant correlation between rhizosphere bacterial diversity and soil C (r = 0.2345, *p* = 0.008), and fungal composition in rhizosphere and non-rhizosphere soil was significantly correlated to soil C and pH. It is widely believed among the scientific community that soil C and soil pH have a direct influence on soil microbial communities [62]. Our findings supported the hypothesis that rhizosphere and non-rhizosphere soil communities from different sub-dams would differ and results from our study support results from other studies [59,63].

Microbes, being the driving factor of biogeochemical cycling, are a crucial ecosystem component. Total C and N content in soil is the key factor that affects microbial biomass and activity [64]. In this study, results from the species richness and Margalef’s richness indices demonstrated that bacteria in the rhizosphere was positively correlated to N content while results from the Shannon–Wiener and evenness indices demonstrated that bacteria in the rhizosphere was significantly and negatively correlated to C content in the S525 sub-dam (Figure 5). In soil, C and N provide energy to microbes and aid in the formation of cell components to maintain microbial subsistence. Furthermore, the soil C/N ratio reflects the biological activities of soil bacterial communities and quantifies mineralization and sequestration characteristics of C and N in soil, which is an important indicator of soil C and N cycling [65,66]. Generally, the C/N ratio is inversely proportional to its decomposition rate [67]. Bacteria are required to input N to satisfy their growth needs when the C/N ratio is high. However, under lower C/N ratios, redundant N from bacterial growth is released into litter and soil [68].

The results from the species richness and Margalef’s richness indices in this study demonstrated that the rhizosphere bacterial community was positively correlated to NO_3_^−^-N and that fungal diversity in non-rhizosphere soil in the S525 sub-dam was to a great extent negatively correlated to NH_4_^+^-N. Moreover, results from the species richness and Margalef’s richness indices found that the rhizosphere bacterial community in the S531 sub-dam was significantly and positively correlated to NH_4_^+^-N in soil and that fungal diversity in the rhizosphere of the S536 sub-dam was significantly correlated to the C/N ratio. Additionally, the fungal community in non-rhizosphere soil was significantly correlated to NO_3_^−^-N, total N, C/N, and Zn (Figure 5), which indicated that the driving factors of microbial composition and diversity in rhizosphere and non-rhizosphere soil varied during restoration times investigated. On the one hand, this may be due to the mineralization and decomposition of soil organic matter and that nutrient transformation differed during the different restoration times [69,70]. In addition, the different C sources could result from how vegetation restoration patterns significantly influence metabolic activities and functional diversity of microbial communities in sandy soil [62]. On the other hand, vegetation production varied in the different sub-dams under different restoration times. Most microorganisms are heterotrophs that require nutrients provided by plants. Soil microorganisms will also decompose organic matter and litter in the case of nutrient available for plant growth [71]. Root exudates could also influence microbial communities in rhizosphere and non-rhizosphere soil [72,73]. These factors indicated that the significant effects that vegetation has on soil microbial communities could be due to differences in litter inputs and root exudates [74,75]. Findings from this study could be used to explore microbial structures and their influencing factors for the ecological restoration of mining areas and also provide a scientific basis to further understand the functional potential of microorganisms.

## 5. Conclusions

In this study, we found that arsenic content decreased gradually as restoration time increased. Total C in shoots and total N in roots of *B. ischaemum* also increased as restoration time increased. In addition, total N in soil was highly and remarkably positively correlated to catalase and urease but highly and significantly negatively correlated to sucrose. Bacterial composition was positively correlated to catalase, sucrase, and phosphatase, and fungal diversity was positively correlated to sucrose in bulk soil. Moreover, restoration time was the main impact factor for bacterial and fungal composition and bacterial diversity and the driving factors of microbial composition and diversity varied in rhizosphere and non-rhizosphere soil for the different restoration times investigated.

## Figures and Tables

**Table 1:** Soil chemical properties of the different sub-dams (Mean ± Standard error). Significant differences between sites (Duncan test, *p* < 0.05) are denoted with letters (a > b > c).

	NH_4_^+^-N mg/kg	NO_3_^+^-N mg/kg	NO_2_^+^-N mg/kg	Olsen-P mg/kg	N mg/kg	C mg/kg	C/N	S mg/kg	SWC %	pH	PS μm
S523	4.566 ± 0.487bc	5.160 ± 0.513	0.350 ± 0.028	6.218 ± 1.339	0.053 ± 0.005	1.089 ± 0.053	21.092 ± 1.563	0.068 ± 0.011	2.222 ± 0.451	7.987 ± 0.075	44.98 ± 4.717
S525	3.974 ± 1.102c	5.782 ± 0.280	0.368 ± 0.045	5.108 ± 1.290	0.045 ± 0.003	1.199 ± 0.102	26.789 ± 1.717	0.062 ± 0.007	1.798 ± 0.552	7.881 ± 0.074	42.60 ± 1.799
S529	7.664 ± 1.318a	5.276 ± 0.739	0.338 ± 0.041	13.428 ± 5.870	0.049 ± 0.007	1.061 ± 0.064	23.191 ± 2.592	0.058 ± 0.007	1.378 ± 0.292	8.052 ± 0.100	42.00 ± 3.933
S531	6.028 ± 0.602abc	6.648 ± 1.497	0.330 ± 0.065	8.682 ± 1.900	0.042 ± 0.006	0.948 ± 0.054	24.279 ± 3.409	0.052 ± 0.004	1.475 ± 0.506	8.111 ± 0.028	40.66 ± 2.410
S536	7.086 ± 0.560ab	5.338 ± 0.744	0.350 ± 0.054	7.970 ± 0.540	0.041 ± 0.005	0.907 ± 0.047	23.349 ± 2.031	0.046 ± 0.001	1.487 ± 0.391	8.023 ± 0.084	46.66 ± 4.829

Abbreviations mean ammonium nitrogen (NH_4_^+^-N), nitrate nitrogen (NO_3_^−^-N), nitrite nitrogen (NO_2_^−^-N), total nitrogen (N), total carbon (C), total sulfur (S), the ratio of carbon and nitrogen (C/N), soil water content (SWC), and average particle size (PS).

**Table 2:** Soil heavy metals of the different sub-dams and transfer factors of different heavy metals in *B. ischaemum* (Mean ± Standard error). The different case letters (a > b > c) indicate that the means are significantly different among reclaimed scenario (*p* < 0.05) with the Duncan test.

	As ppm	Cd ppm	Cu ppm	Pb ppm	Zn ppm	TF-Cd	TF-Cr	TF-Cu	TF-Pb	TF-Zn
S523	10.318 ± 2.783b	6.798 ± 0.620	366.445 ± 20.368	258.116 ± 24.396	81.005 ± 8.800	0.726 ± 0.169	0.201 ± 0.065	0.474 ± 0.107	1.530 ± 0.654	1.289 ± 0.285
S525	10.843 ± 2.180b	7.258 ± 0.747	379.141 ± 30.697	250.481 ± 36.774	81.040 ± 5.049	0.426 ± 0.173	0.131 ± 0.104	0.289 ± 0.084	0.830 ± 0.159	0.743 ± 0.286
S529	13.183 ± 3.013b	7.469 ± 0.382	324.415 ± 17.770	277.673 ± 29.442	85.061 ± 5.447	0.664 ± 0.092	0.449 ± 0.126	0.514 ± 0.096	1.177 ± 0.169	1.045 ± 0.168
S531	12.190 ± 5.102b	7.111 ± 1.159	326.789 ± 34.125	261.731 ± 19.734	87.296 ± 11.009	0.612 ± 0.142	0.410 ± 0.175	0.491 ± 0.158	1.100 ± 0.183	0.992 ± 0.272
S536	25.440 ± 3.003a	6.309 ± 0.831	352.808 ± 41.025	224.320 ± 33.932	69.542 ± 6.377	0.576 ± 0.052	0.481 ± 0.130	0.659 ± 0.131	1.036 ± 0.265	0.876 ± 0.087

**Table 3:** The Pearson correlations between chemical properties of soil, root and shoot, soil enzyme, heavy metals and transfer factors in the copper tailings dam.

		Soil Enzyme Activity				Soil Heavy Metals					Transfer Factors				
		Catalase	Urease	Sucrase	Phosphatase	As	Cd	Cu	Pb	Zn	TF-Cd	TF-Cr	TF-Cu	TF-Pb	TF-Zn
Soil	NH_4_^+^-N	0.036	0.224	0.157	−0.055	0.232	−0.088	−0.250	0.025	0.022	−0.057	0.261	0.095	−0.158	−0.167
	NO_3_^+^-N	0.232	0.209	−0.277	0.269	−0.368	−0.286	−0.282	−0.005	0.400 *	−0.128	0.108	0.166	−0.292	−0.231
	NO_2_^+^-N	−0.011	−0.083	0.07	−0.133	0.191	−0.147	0.280	−0.269	−0.286	−0.113	−0.204	0.082	0.020	0.067
	Olsen-P	−0.003	−0.02	−0.153	0.017	0.114	0.113	−0.174	0.162	0.071	0.113	0.354	0.354	−0.016	−0.083
	N	0.722 **	0.695 **	−0.650 **	0.228	−0.652 **	−0.137	−0.323	0.147	0.316	−0.021	−0.079	0.029	0.013	0.212
	C	0.327	0.29	−0.199	−0.023	−0.494 *	0.167	0.238	0.005	0.200	0.125	−0.019	0.042	0.229	0.384
	C/N	−0.581 **	−0.530 **	0.668 **	−0.286	0.425 *	0.324	0.580 **	−0.112	−0.263	0.125	0.125	−0.019	0.140	0.101
	S	0.083	0.195	−0.335	0.010	−0.202	0.023	0.231	−0.100	0.142	0.346	0.002	0.284	0.682 **	0.376
	SWC	−0.036	−0.232	0.237	−0.164	0.124	−0.043	0.449 *	−0.134	−0.159	0.474*	0.141	0.004	0.118	0.167
	pH	−0.002	0.297	−0.041	0.255	0.043	0.298	−0.208	−0.112	0.013	−0.190	−0.084	−0.231	0.004	0.277
	PS	−0.309	−0.256	0.167	−0.142	0.385	−0.229	0.199	0.091	−0.028	−0.180	−0.182	−0.068	−0.103	−0.285
Shoot	N	−0.088	−0.368	0.227	−0.218	0.274	0.012	0.232	−0.476 *	−0.209	0.363	−0.026	−0.163	−0.082	−0.092
	C	0.150	−0.079	−0.207	−0.204	−0.247	−0.114	0.134	0.084	−0.120	−0.092	−0.603 **	−0.337	−0.120	−0.135
	C/N	0.173	0.430 *	−0.295	0.166	−0.344	−0.109	−0.321	0.535 **	0.167	−0.308	−0.093	0.129	0.078	0.063
	S	0.028	0.348	−0.158	−0.119	−0.322	0.066	−0.092	0.139	0.358	−0.161	0.145	0.008	0.080	0.125
Root	N	0.307	−0.127	−0.021	0.162	−0.211	−0.018	−0.137	−0.082	0.059	0.266	−0.066	−0.170	−0.337	−0.205
	C	0.140	−0.191	−0.230	−0.628 **	0.192	−0.263	0.276	0.078	−0.475 *	0.353	0.097	0.422	0.164	0.156
	C/N	−0.294	0.08	−0.076	−0.289	0.223	−0.061	0.189	0.114	−0.201	−0.076	0.027	0.242	0.501 *	0.309
	S	−0.406	−0.207	0.137	−0.446 *	0.330	0.075	0.170	−0.139	−0.122	0.248	0.538 **	0.335	0.187	0.069

Abbreviations: NH_4_^+^-N, ammonium nitrogen, NO_3_^−^-N, nitrate nitrogen, NO_2_^−^-N, nitrite nitrogen, N, total nitrogen, C, total carbon, S, total sulfur, C/N, the ratio of carbon and nitrogen, SWC, soil water content, and PS, average particle size. ** Correlation is significant at the 0.01 level (2-tailed). * Correlation is significant at the 0.05 level (2-tailed).

**Table 4:** Two-way ANOVA of microbial diversity and microbial gene abundances of rhizosphere and non-rhizosphere soil in different sub-dams.

		df	*H’*		*S*		*dMa*		*En*		*D*		logCopy	
			F	*p*	F	*p*	F	*p*	F	*p*	F	*p*	F	*p*
Bacteria	Restoration years (Y)	4	26.307	**<0.01**	124.138	**<0.01**	191.302	**<0.01**	28.332	**<0.01**	12.129	**<0.01**	25.906	**<0.01**
	Rhizosphere region (R)	1	1.881	0.178	0.929	0.341	2.529	0.120	0.023	0.88	3.097	0.086	121.932	**<0.01**
	Y × R	4	5.598	**<0.01**	4.137	**<0.01**	2.640	**0.048**	1.118	0.361	2.951	**0.032**	17.512	**<0.01**
Fungi	Restoration years (Y)	4	5.858	**<0.01**	5.396	**<0.01**	4.632	**<0.01**	0.837	0.510	6.089	**<0.01**	3.996	**<0.01**
	Rhizosphere region(R)	1	7.483	**<0.01**	6.366	**0.016**	2.544	0.119	1.304	0.260	8.151	**<0.01**	414.610	**<0.01**
	Y × R	4	1.070	0.384	0.682	0.609	0.866	0.493	1.017	0.410	1.633	0.185	2.526	0.056

Abbreviations: Shannon-Wiener index (*H’*), Species richness (*S*), Margalef (*d_Ma_*), Evenness (*E_n_*) and Simpson index (*D*), ratio of bacteria and fungi gene copies (B/F). Significant *p*-values are in bold print.

**Table 5:** Relationships of microbial compositions and diversities to different soil enzyme activities by a Mantel test.

		Catalase		Urease		Sucrase		Phosphatase	
		rM	*p*	rM	*p*	rM	*p*	rM	*p*
Rhizosphere	Bacterial composition	0.1413	0.096	0.15340	0.053	0.09360	0.105	0.11460	0.172
	Fungal composition	−0.1320	0.914	−0.04207	0.652	0.02596	0.357	−0.02459	0.564
	Bacterial diversity	−0.1435	0.907	−0.07609	0.739	0.1285	0.056	−0.15320	0.934
	Fungal diversity	−0.1065	0.851	−0.1058	0.863	0.03853	0.217	−0.05151	0.658
Non-rhizosphere	Bacterial composition	0.2178	**0.024**	0.16830	0.064	0.1573	**0.026**	0.27940	**0.008**
	Fungal composition	−0.1320	0.932	−0.04207	0.675	0.02596	0.343	−0.02459	0.620
	Bacterial diversity	−0.06443	0.664	0.01617	0.412	0.1481	0.065	0.001121	0.415
	Fungal diversity	−0.09926	0.731	0.07597	0.236	0.2057	**0.024**	−0.02155	0.428

Note: Significant *p*-values are in bold print.

**Table 6:** Relationships of microbial compositions and diversities to soil and root variables, restoration years and transfer factors by Mantel test.

		Soil Enzyme Activity		Soil Heavy Metals		Root Properties		Root Heavy Metals		Restoration Years		Transfer Factors	
		*rM*	*p*	*rM*	*p*	*rM*	*p*	*rM*	*p*	*rM*	*p*	*rM*	*p*
Rhizosphere	Bacterial composition	−0.03053	0.619	−0.14950	0.941	−0.0348	0.647	0.00050	0.498	0.3124	**0.001**	0.1149	0.127
	Fungal composition	−0.00690	0.548	0.10840	0.101	−0.0487	0.717	−0.02232	0.592	0.3052	**0.001**	−0.1714	0.985
	Bacterial diversity	−0.05227	0.720	0.07106	0.198	−0.2032	0.990	−0.04485	0.690	0.3417	**0.001**	−0.1207	0.893
	Fungal diversity	−0.03539	0.652	−0.05688	0.764	0.02572	0.362	−0.08115	0.807	0.01618	0.354	0.05851	0.262
Non-rhizosphere	Bacterial composition	0.02491	0.408	0.00248	0.504	0.1211	0.114	0.07338	0.200	0.4749	**0.001**	0.1037	0.169
	Fungal composition	−0.00690	0.516	0.10840	0.093	−0.0487	0.714	−0.02232	0.584	0.3052	**0.001**	−0.1714	0.985
	Bacterial diversity	−0.04229	0.610	0.01901	0.438	−0.1590	0.939	−0.05650	0.659	0.3812	**0.001**	−0.0217	0.532
	Fungal diversity	0.02797	0.360	−0.1138	0.806	−0.1367	0.878	−0.09095	0.719	0.1195	0.063	−0.0440	0.542

Note: Significant *p*-values are in bold print.
